# The improvement of photocatalytic activity of monolayer g-C_3_N_4_*via* surface charge transfer doping

**DOI:** 10.1039/c7ra12444a

**Published:** 2018-01-09

**Authors:** F. L. Yang, F. F. Xia, J. Hu, C. Z. Zheng, J. H. Sun, H. B. Yi

**Affiliations:** School of Chemical and Environmental Engineering, Jiangsu University of Technology Changzhou 213001 Jiangsu P. R. China; State Key Laboratory of Chemo/Biosensing and Chemometrics, College of Chemistry and Chemical Engineering, Hunan University Changsha 410082 Hunan P. R. China ffxia@jsut.edu.cn sunjh@jsut.edu.cn hbyi@hnu.edu.cn

## Abstract

Graphite-like carbon nitride (g-C_3_N_4_) has attracted much attention due to its peculiar photocatalytic performance as a visible-light-responsive photocatalyst. However, its insufficient sunlight absorption is not conducive to the photocatalytic activity of the g-C_3_N_4_. Herein, by using first-principles density functional theory (DFT) calculations, we demonstrated a simple yet efficient way to achieve improvement of photocatalytic activity of monolayer g-C_3_N_4_*via* surface charge transfer doping (SCTD) using the electron-drawing tetracyanoquinodimethane (TCNQ) and electron-donating tetrathiafulvalene (TTF) as surface dopants. Our calculations revealed that the electronic properties of monolayer g-C_3_N_4_ can be affected by surface modification with TCNQ and TTF. These dopants are capable of drawing/donating electrons from/to monolayer g-C_3_N_4_, leading to the accumulation of holes/electrons injected into the monolayer g-C_3_N_4_. Correspondingly, the Fermi levels of monolayer g-C_3_N_4_ were shifted towards the valence/conduction band regions after surface modifications with TCNQ and TTF, along with the increase/decrease of work functions. Moreover, the optical property calculations demonstrated that the TCNQ and TTF modifications could significantly broaden the optical absorption of monolayer g-C_3_N_4_ in the visible-light regions, yielding an improvement in the photocatalytic activity of monolayer g-C_3_N_4_. Our results unveil that SCTD is an effective way to tune the electronic and optical properties of monolayer g-C_3_N_4_, thus improving its photocatalytic activity and broadening its applications in splitting water and degrading environmental pollutants under sunlight irradiation.

## Abstract 2

The improvement of optical adsorption for monolayer g-C_3_N_4_*via* surface charge transfer doping.

## Graphical abstract

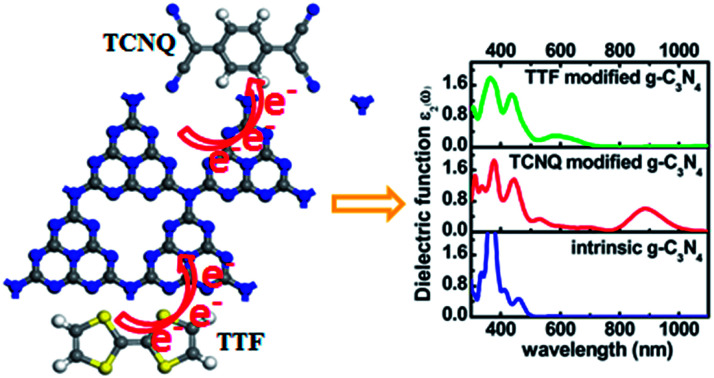

## Introduction

Graphitic carbonic nitride (g-C_3_N_4_) has attracted much attention since it was first developed to be a visible-light-driven photocatalyst by Wang and co-workers in 2009 due to its abundance, high stability, and excellent capacity for solar utilization.^1^ Therefore g-C_3_N_4_ has been found to be important in applications in diverse fields,^1–11^ including water splitting,^1,2^ CO_2_ reduction,^3,4^ contaminant degradation,^5,6^ and so on. Unfortunately, bulk g-C_3_N_4_ is a medium band gap semiconductor with visible light response (up to 450 nm), but low carrier mobility and insufficient sunlight absorption limit its photocatalytic activity,^1,8–11^ which cannot meet the prerequisites of high activity in photocatalytic reaction (both strong light absorption and suitable redox potential).^12,13^ Therefore, there are many reports on the improvement of photocatalytic activity for pristine g-C_3_N_4_.^14–32^ For instance, Ma *et al.* proposed an effective structural doping approach to modify the photoelectrochemical properties of g-C_3_N_4_ by doping with nonmetal (sulfur or phosphorus) impurities, which reduced the energy gap to enhance the visible-light absorption of g-C_3_N_4_.^16^ Lu *et al.* reported that the photocatalytic efficiency of g-C_3_N_4_ can be enhanced by H ions plus B, N, Si, O, P and As ions with high coverage rates plus halogen ions with low coverage rates.^17^ Although much effort has been devoted to improving the photocatalytic activity of g-C_3_N_4_, it shows great necessity and urgency to discover new efficient approach to overcome the above mentioned problems and broaden the applications of g-C_3_N_4_ in water splitting and environmental pollutants degradation fields.

It has been reported that element doping is an efficient method to tune the unique electronic structure and band gap of g-C_3_N_4_, which considerably broadens the light responsive range and enhance the charge separation.^16,17,20^ However, the conventional doping with elemental impurities methods usually can introduce any bulk defects into the semiconductor lattice. In contrast, the surface charge transfer doping (SCTD) approach is nondestructive and does not induce any bulk defects into the semiconductor lattice, thus retaining the high performance of nanostructures by reducing carrier scattering in the bulk.^33–35^ In this approach, through controlling Fermi level (*E*_F_) misalignment of surface dopants with respect to underlying semiconductor nanostructures, electrons can be extracted from (or injected into) the nanostructures, forming an electron-deficient (or electron-rich) surface layer. Carrier concentration and even conduction type of the semiconductor nanostructures can be readily tuned by varying the types as well as densities of surface dopants, leading to effective p- and n-type doping on the nanostructures.^33–35^ SCTD has been proven to be a simple, nondestructive, and effective method to tune both the electronic and optical properties of low-dimensional semiconductors,^36–44^ which is of fundamental importance to enable their wide applications in optoelectronic and electronic devices. For example, the electronic properties and carrier density of monolayer MoS_2_ monolayer could be effectively modulated with electron acceptor, tetracyanoquinodimethane (TCNQ), and electron donor, tetrathiafulvalene (TTF).^41^ Zhang *et al.*^44^ showed that phosphorene could be p- and n-type doped by modifying the surface with TCNQ and TTF, respectively. On the other hand, it was also reported that the optical properties of two dimensional (2D) materials could be modulated by SCTD. Jing *et al.*^41,43^ disclosed that TCNQ and TTF could enhance the optical properties of MoS_2_ and phosphorene for effective light harvesting. All these reports suggested that SCTD is of fundamental importance to broaden their applications in optoelectronic and electronic devices. Although the SCTD scheme has been demonstrated to be very effective for MoS_2_ and phosphorene, so far there are few works that apply this method on the monolayer g-C_3_N_4_.^28,29^

Herein, based on the density functional theory (DFT) with first-principles calculations, we demonstrated an efficient approach to improve the photocatalytic activity of monolayer g-C_3_N_4_ by SCTD using TCNQ and TTF as surface dopants. Our calculations revealed that TCNQ and TTF could act as acceptor and donor to inject holes and electrons into monolayer g-C_3_N_4_, leading to electron-deficient and -rich surface layers, respectively. The remarkable surface charge transfer between the adsorbed molecules and the monolayers made TCNQ/TTF an efficient surface dopant to rationally tune the electronic and optical properties of monolayer g-C_3_N_4_. For TCNQ and TTF modified systems, the Fermi levels moves into the valence/conduction band region, together with the increase/decrease of work functions, thus leading to p-/n-type doping of monolayer g-C_3_N_4_. Moreover, the SCTD with TCNQ and TTF has also been found to be an effective way to enhance the light harvesting capabilities of the monolayer g-C_3_N_4_ in the visible-light region, which improves the photocatalytic activity and broadens the applications in splitting water and degrading environmental pollutants under sunlight irradiation.

## Computational methods

All the first-principles calculations were performed using DFT based methods as implemented in the Cambridge Sequential Total Energy Package (CASTEP) program in Materials Studio 6.1 package of Accelrys Ltd.^45–47^ The Generalized Gradient Approximation (GGA)^48^ with the Perdew–Burke–Ernzerhof functional (PBE)^49,50^ was adopted to describe the correction of the electronic exchange and correlation effects. Meanwhile, the van der Waals interactions between the monolayers and surface dopants (TCNQ and TTF) were described by the DFT-D2 method of Grimme.^51^ The interactions between valence electrons and ionic core were described by the Vanderbilt ultrasoft pseudopotential.^52^ The cutoff energy was set as 550 eV, and 8 × 8 × 1 *k*-points with the Monkhorst–Pack^53^ scheme in the first Brillouin zone was employed in the present work. Both the cutoff energy and *k* grid were tested to be converged in the total energy. A slab of vacuum of 15 Å in thickness in the *Z* direction was applied to avoid the interaction with the image atoms. All of the structure models were fully relaxed, and the convergence criteria for geometric optimization and energy calculation were set to 2.0 × 10^−5^ eV per atom, 0.02 eV Å^−1^, 0.005 Å and 2.0 × 10^−6^ eV per atom for the tolerance of energy, maximum force, maximum ionic displacement, and self-consistent field (SCF), respectively.

In addition, the available adsorption sites of TCNQ and TTF on the surface of monolayer g-C_3_N_4_ were explored by comparing the adsorption energy (Δ*E*). And the Δ*E* was calculated according to the following definition:Δ*E* = *E*_dopant/g-C_3_N_4__ − *E*_g-C_3_N_4__ − *E*_dopant_where *E*_dopant/g-C_3_N_4__, *E*_g-C_3_N_4__, and *E*_dopant_ are the total energy of the surface modified system, intrinsic monolayer g-C_3_N_4_, and isolated dopant, respectively.

## Results and discussion

**Fig. 1:**
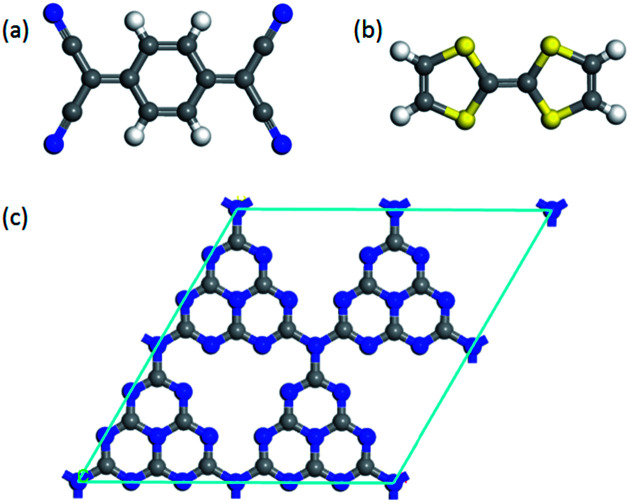
The optimized energetically most favorable configurations for top views of (a) TCNQ, (b) TTF and (c) monolayer g-C_3_N_4_.

**Fig. 2:**
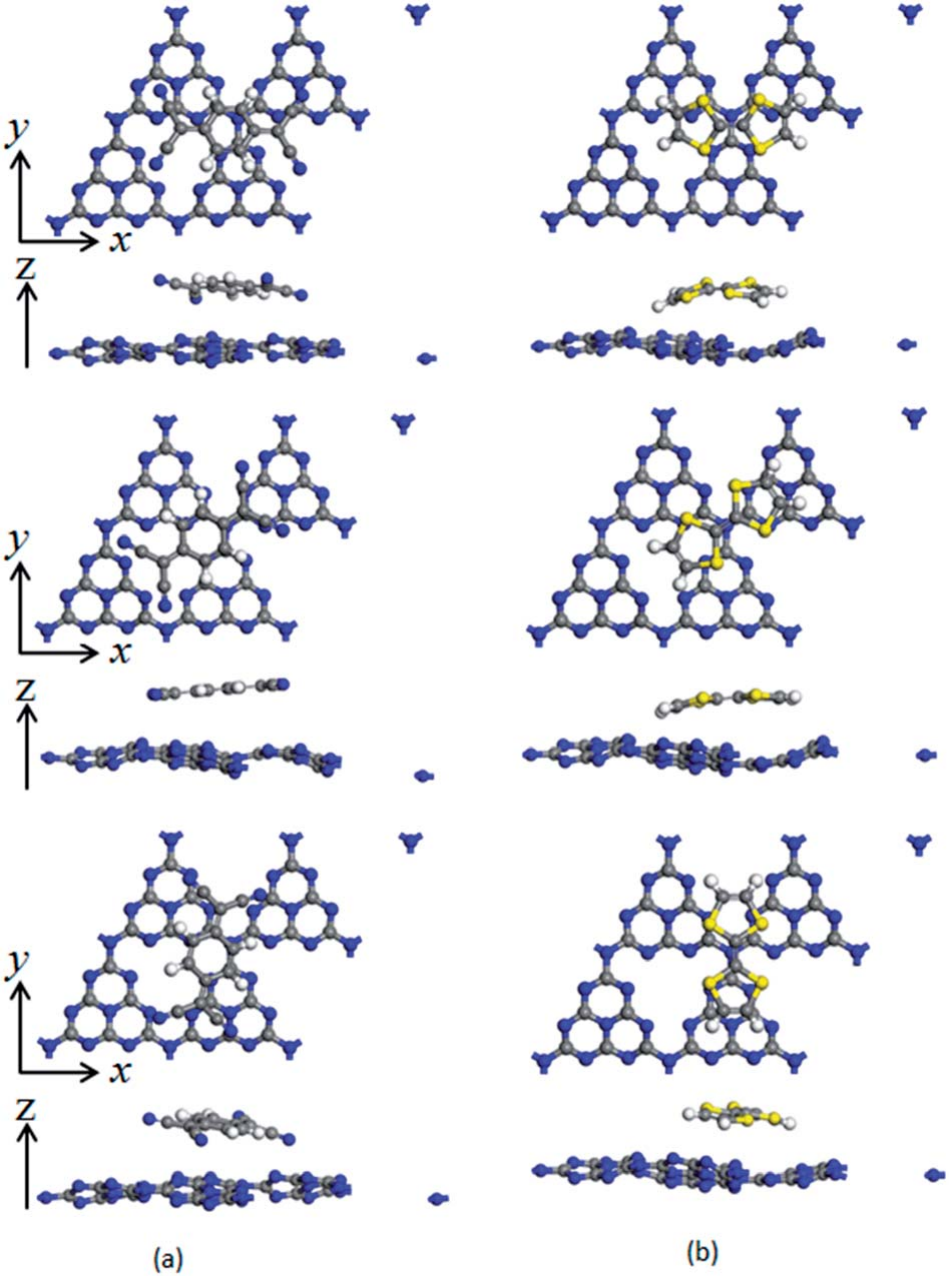
Top and side views of TCNQ (a) and TTF (b) modified monolayer g-C_3_N_4_. Three adsorption positions on the surface of monolayer g-C_3_N_4_ are considered, and the adsorption positions along with *z* axis are 0°, 45° and 90° corresponding to the first line, second line and third line, respectively.

To investigate the surface charge transfer doping effects on the monolayer g-C_3_N_4_, two typical p- and n-type organic surface dopants, including one electron-withdrawing molecule (TCNQ) and one electron-donating molecule (TTF), were chosen in this paper, as shown in Fig. 1a and b. And Fig. 1c also presents the top views of the energetically most favorable configuration of monolayer g-C_3_N_4_. For each molecule, we considered several possible adsorption sites and the energetically favorable configuration for these two molecules on the monolayer g-C_3_N_4_ are presented in Fig. 2. And the possible adsorption sites of TCNQ and TTF on the surface of monolayer g-C_3_N_4_ were explored by comparing the adsorption energy (Δ*E*), which was defined in the computational method section and a more negative Δ*E* indicated a more favorable configuration.

The electron-withdrawing/donating molecules, TCNQ and TTF, prefer adsorption on the basal surface of monolayer g-C_3_N_4_ along the *z* direction with a vertical distance. And the adsorption energy of the three different configurations of TCNQ and TTF modified systems is −0.12, −0.11, −0.12, −0.12, −0.12 and −0.12 eV (Table 1), respectively, showing a non-covalent interaction between the surface dopants and monolayer g-C_3_N_4_. It can be noted that the structure of monolayer g-C_3_N_4_ was obviously bending after TCNQ and TTF surface modification due to the non-covalent interaction between the surface dopants and monolayer g-C_3_N_4_. In addition, Mulliken charge analysis of bond population^54,55^ for the TCNQ modified systems show that there are about −0.11 charge transfers from the monolayer g-C_3_N_4_ to TCNQ, revealing that the TCNQ molecule can act as a strong acceptor on the surface of monolayer g-C_3_N_4_. In contrast, for the TTF modified monolayer g-C_3_N_4_ systems, TTF acts as a donor and injects electrons into the monolayers (as shown in Table 1). As a result, the adsorption of TCNQ and TTF lead to positively and negatively charged monolayer g-C_3_N_4_, manifesting that surface modification by TCNQ and TTF molecules could be an effective approach to modulate the carrier concentrations in g-C_3_N_4_ monolayers.

**Table 1:** Adsorption energy (Δ*E*), charge transfer (*q*) and change of work function (Δ*Φ*)^a^ of the TCNQ and TTF modified monolayer g-C_3_N_4_

Geometries	Position	Δ*E*/eV	*q*_ads_/e	*q*_g-C_3_N_4__/e	Δ*Φ*/eV
TCNQ modified g-C_3_N_4_	0°	−0.12	−0.12	0.12	1.21
	45°	−0.11	−0.10	0.10	1.20
	90°	−0.12	−0.11	0.11	1.19
TTF modified g-C_3_N_4_	0°	−0.12	0.20	−0.30	−0.30
	45°	−0.12	0.19	−0.29	−0.17
	90°	−0.12	0.22	−0.22	−0.13

a Δ*Φ* is defined as Δ*Φ* = *Φ*_dopant/g-C_3_N_4__ − *Φ*_g-C_3_N_4__, where *Φ*_dopant/g-C_3_N_4__ and *Φ*_g-C_3_N_4__ are the work functions of the surface modified system and the intrinsic monolayer g-C_3_N_4_, respectively.

**Fig. 3:**
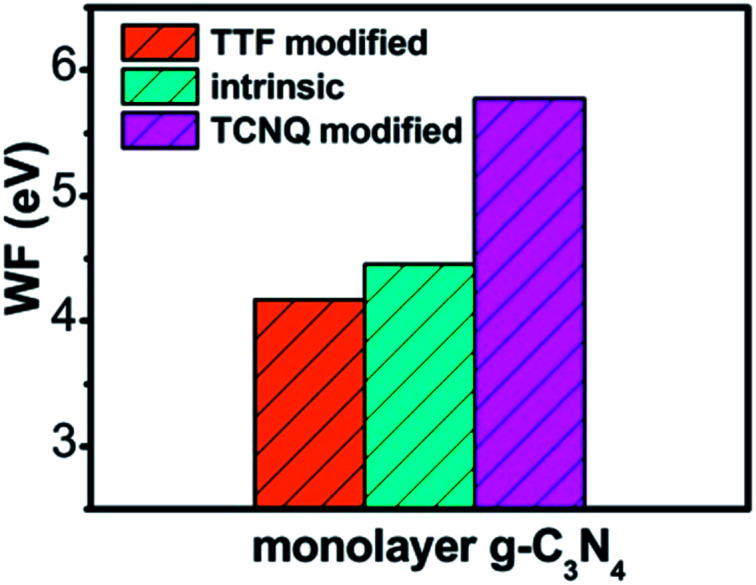
The plot of calculated work function of intrinsic and different organic molecules modified monolayer g-C_3_N_4_ at GGA/PBE level.

To explore the work function variations of monolayer g-C_3_N_4_ before and after surface modification, the electrostatic potential calculations were further performed to study the changes of work functions (Δ*Φ* = *Φ*_dopant/g-C_3_N_4__ − *Φ*_g-C_3_N_4__) for monolayer g-C_3_N_4_ after surface modifications. As shown in Table 1, there is an obvious increase of work functions for monolayer g-C_3_N_4_ by 1.21, 1.20, and 1.19 eV, respectively, after the adsorption of TCNQ, attributing to the injection of holes from TCNQ into the monolayers. In contrast, due to the injection of electrons from TTF molecule, the adsorption of TTF results in a decrease of work function by 0.30, 0.17, and 0.13 eV, respectively. It can be also noted that the work function of TCNQ modified monolayer g-C_3_N_4_ is higher than that of intrinsic monolayers, while the work function of TTF modified monolayer g-C_3_N_4_ is lower than that of intrinsic monolayers (shown in Fig. 3), in accordance with the literature reports.^40,56^ These phenomena indicate that the monolayer g-C_3_N_4_ may be tuned into p/n-type materials by doping with the electron-withdrawing/donating TCNQ and TTF molecules.

**Fig. 4:**
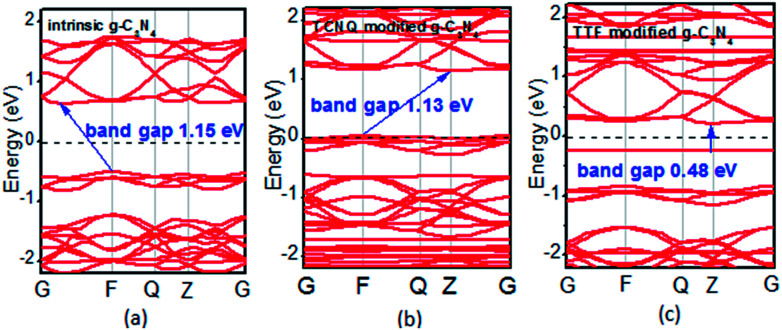
Band structures of (a) intrinsic, (b) TCNQ and (c) TTF modified monolayer g-C_3_N_4_ at GGA/PBE level. The dashed lines indicate the *E*_F_.

**Fig. 5:**
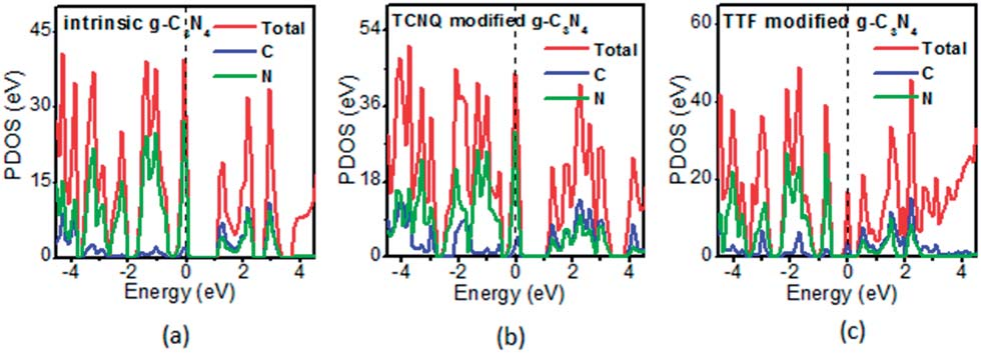
The projected density of states (PDOS) of the (a) intrinsic, (b) TCNQ and (c) TTF modified monolayer g-C_3_N_4_ at GGA/PBE level. The dashed lines indicate the *E*_F_.

To examine whether the surface dopants (TCNQ and TTF) have an effect on electronic properties of the monolayer g-C_3_N_4_ due to charge transfer between surface dopants (TCNQ and TTF) and monolayer g-C_3_N_4_, the electronic band structures of monolayer g-C_3_N_4_ before and after surface modification were computed (Fig. 4). Fig. 4a–c presents the electronic band structures of the intrinsic and surface modified monolayer g-C_3_N_4_, respectively. Remarkably, the intrinsic monolayer g-C_3_N_4_ is an indirect-gap semiconductor with a bandgap of 1.15 eV at GGA/PBE level, while with a bandgap of 2.70 eV at HSE06 level, which agrees well with previous reports.^24,57^ For TCNQ modified system, it can be noted that the Fermi level (*E*_F_) moves into the valence band region that probably attributed to the adsorption of TCNQ molecule. And the shift of original *E*_F_ in monolayer g-C_3_N_4_ to the lower energy regions leads to the increase of work function. These phenomena reveal that monolayer g-C_3_N_4_ can be p-type doped with TCNQ surface modification. In contrast, for TTF modified system, there appears a new flat energy level below the Fermi level that mainly attributed to the TTF molecule. The appearance of the new empty flat band in the bandgaps shifts the original *E*_F_ in the monolayer g-C_3_N_4_ to the higher energy region, thus decreases the work functions. The new flat level can act as a donor state in TTF-modified system, which is in favor of the charge transfer from TTF to monolayer g-C_3_N_4_. In the meantime, the new flat band below the Fermi level is close to the conduction band minimum (CBM) in the TTF modified system (Fig. 4c), verifying that it acts as a donor state for n-type doping. The density of states (DOS) of the molecule-modified systems and the projected density of states (PDOS) for both the adsorbed molecules and monolayer g-C_3_N_4_ in these adsorption systems were also computed (Fig. 5), which validate the results from the band structures and confirm that the new flat energy levels were generated by the absorbed molecule. The above results collectively demonstrate that TCNQ and TTF can p- and n-type doping of monolayer g-C_3_N_4_, respectively.

**Fig. 6:**
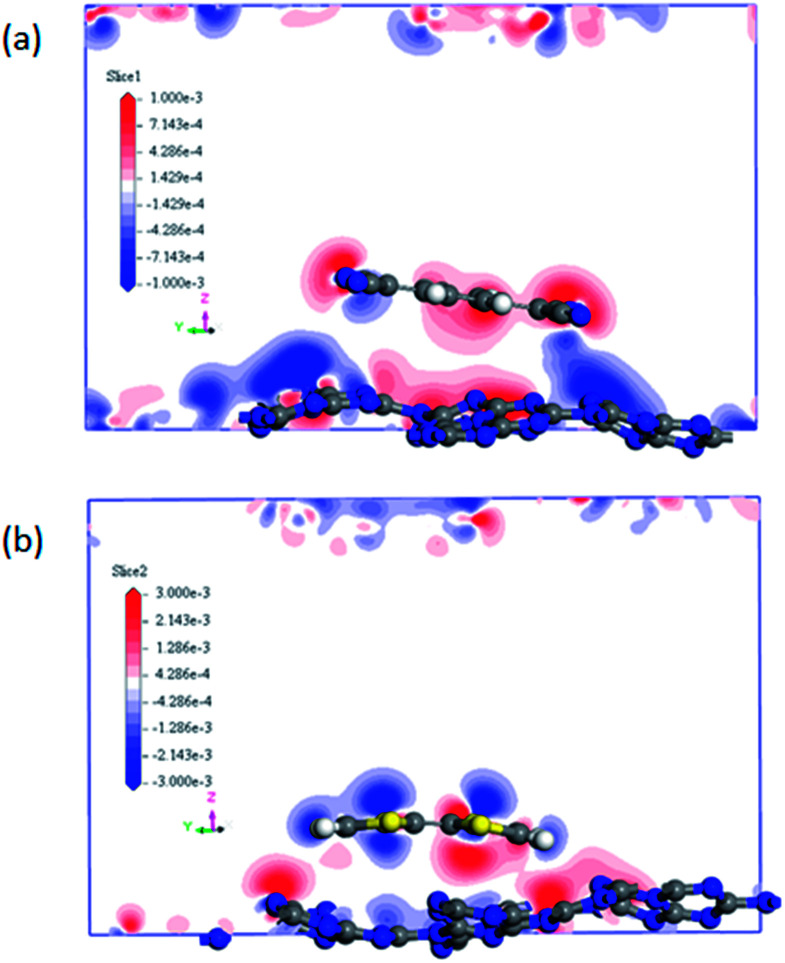
The difference electron density of (a) TCNQ and (b) TTF modified monolayer g-C_3_N_4_. A loss of electrons is indicated in blue, while electron enrichment in red.

It can be noted that the charge transfer between the surface dopants (TCNQ and TTF) and monolayer g-C_3_N_4_ have an obvious effects on the electronic properties of monolayer g-C_3_N_4_. Therefore, the electron density difference (Δ*ρ*) of monolayer g-C_3_N_4_ before and after TCNQ and TTF modification was calculated to visualize the charge transfer between the surface dopants (TCNQ and TTF) and monolayer g-C_3_N_4_ (Fig. 6). Δ*ρ* illustrates how the electron density changes during the adsorption process and is defined as Δ*ρ* = *ρ*_dopant/g-C_3_N_4__ − *ρ*_dopant_ − *ρ*_g-C_3_N_4__, in which *ρ*_dopant/g-C_3_N_4__, *ρ*_dopant_, and *ρ*_g-C_3_N_4__ denote the electron density of the surface modified system, the isolated molecule and the isolated monolayer g-C_3_N_4_, respectively. Fig. 6a and b show the change of electron density in the monolayer g-C_3_N_4_ after TCNQ and TTF surface modification, where the gain and loss of electrons is presented in red and blue color, respectively. For TCNQ modified system, there are obvious electrons depletion on the surface of monolayer g-C_3_N_4_, while strong electrons accumulation around TCNQ molecule. In contrast, for TTF modified system, the adsorption of TTF leads to the electrons enrichment on the surface of monolayer g-C_3_N_4_ and the electrons depletion around TTF molecule. These phenomena intuitively reveal that TCNQ and TTF can draw and donate electrons from/to monolayer g-C_3_N_4_ as acceptor and donor, respectively, and these results are consistent with the Mulliken charge transfer analysis in Table 1. In addition, both electrons accumulation and depletion appear on the surface of monolayer g-C_3_N_4_, suggesting there are charge transfers both between intramolecules and intermolecules.

**Fig. 7:**
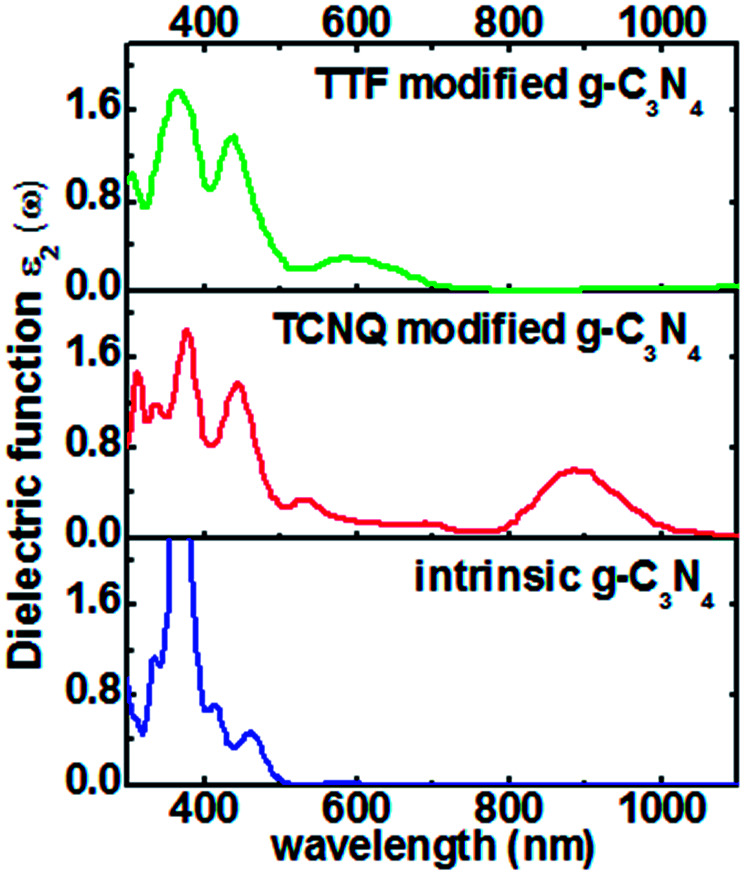
Computed imaginary dielectric functions *versus* wavelength for intrinsic (blue lines), TCNQ (red lines) and TTF (green lines) modified monolayer g-C_3_N_4_.

The electronic properties of monolayer g-C_3_N_4_ can be tuned by the TCNQ and TTF surface modification, and to explore surface modification with TCNQ and TTF whether can improve the optical properties of monolayer g-C_3_N_4_, the optical spectra of the intrinsic and surface-modified monolayer g-C_3_N_4_ were calculated (Fig. 7). The imaginary part of the dielectric functions (*ε*_2_) is an effective parameter to measure the optical absorption ability of materials, and the peaks in *ε*_2_ are caused by the absorption of incident photons and the interband transition of electrons.^58,59^ As shown in Fig. 7, there is an appreciable largest absorption peak around 460 nm for intrinsic monolayer g-C_3_N_4_ (blue curve in Fig. 7), which is in good agreement with previous report^16^ and denotes the monolayer g-C_3_N_4_ is a visible-light semiconductor material. However, the absorption intensity of monolayer g-C_3_N_4_ is weak in the visible-light region, which is not enough to contribute to the highly photocatalytic activity of g-C_3_N_4_. For TCNQ modified system, new absorption peaks appear in the region of 530–880 nm, and the absorption intensity of peak around 880 nm is increased compared to that of intrinsic monolayer g-C_3_N_4_ peak around 460 nm. These new peaks round 530–880 nm could contribute to improve the photocatalytic activity of monolayer g-C_3_N_4_. Similar to TTF modified system, it can be noted that a new visible-light absorption peak appears around 586 nm, which can also contribute to improve the photocatalytic activity of monolayer g-C_3_N_4_. All these phenomena suggest that both TCNQ and TTF surface modification can induce an increase of optical absorption range and the absorption intensity in the visible-light region of monolayer g-C_3_N_4_. This will be of great importance to improve the photocatalytic activity of monolayer g-C_3_N_4_ and broaden its applications in splitting water and degrading environmental pollutants under sunlight irradiation.

## Conclusions

In conclusion, we have proposed an efficient approach to improve the photocatalytic activity of monolayer g-C_3_N_4_*via* surface charge transfer doping with TCNQ and TTF molecules. The electronic and optical properties of intrinsic and surface modified monolayer g-C_3_N_4_ were systematically investigated by means of DFT computations. It was found that TCNQ could act as an acceptor to draw electrons from the monolayer g-C_3_N_4_, leading to pronounced holes accumulation in the monolayers and increased work functions. While TTF could act as a donor to inject electrons into the monolayer g-C_3_N_4_, resulting in the accumulation of electrons on the monolayers and decreased work functions. The remarkable surface charge transfer between the adsorbed molecules and the monolayers made TCNQ/TTF an efficient surface dopant to rationally tune the electronic properties of monolayer g-C_3_N_4_. Moreover, the adsorptions of TCNQ and TTF on monolayer g-C_3_N_4_ could induce an increase of optical absorption range and the absorption intensity in the visible-light region, improving the photocatalytic activity of monolayer g-C_3_N_4_. Our work demonstrates the great potential of SCTD method on the rational tuning of the electronic and optical properties of monolayer g-C_3_N_4_, opening up the opportunities to improve the photocatalytic activity of monolayer g-C_3_N_4_*via* SCTD.

## Conflicts of interest

There are no conflicts to declare.

## Supplementary Material
